# Supplementary material for: Structural basis for DNA break sensing by human MRE11-RAD50-NBS1 and its regulation by telomeric factor TRF2
Source: Nat Commun. 2025 Sep 18;16:8320. doi: 10.1038/s41467-025-64082-x (PMC12446458; doi:10.1038/s41467-025-64082-x)
Supplement: Supplementary file 1 — Supplementary Information [file 41467_2025_64082_MOESM1_ESM.pdf]

## Supplementary Figures and Information of the Manuscript:

### Structural Basis for DNA Break Sensing by Human MRE11-RAD50-NBS1 and its Regulation by Telomeric Factor TRF2

Yilan Fan[1,†], Filiz Kuybu[1,†], Hengjun Cui[1,†], Katja Lammens[1], Jia-Xuan Chen[2], Michael Kugler[1], Christophe Jung[1], Karl-Peter Hopfner[1,*]

[1]Gene Center, Department of Biochemistry, Ludwig-Maximilians-Universität München, Feodor Lynen Straße 25, 81377 Munich, Germany
[2]Proteomics Core Facility, Institute of Molecular Biology, Mainz, Germany
[†]These authors contributed equally
*To whom the correspondence should be addressed. Tel: +49 (0)89 – 2180 76953; Email: karlpeter.hopfner@lmu.de

## Supplementary Figures

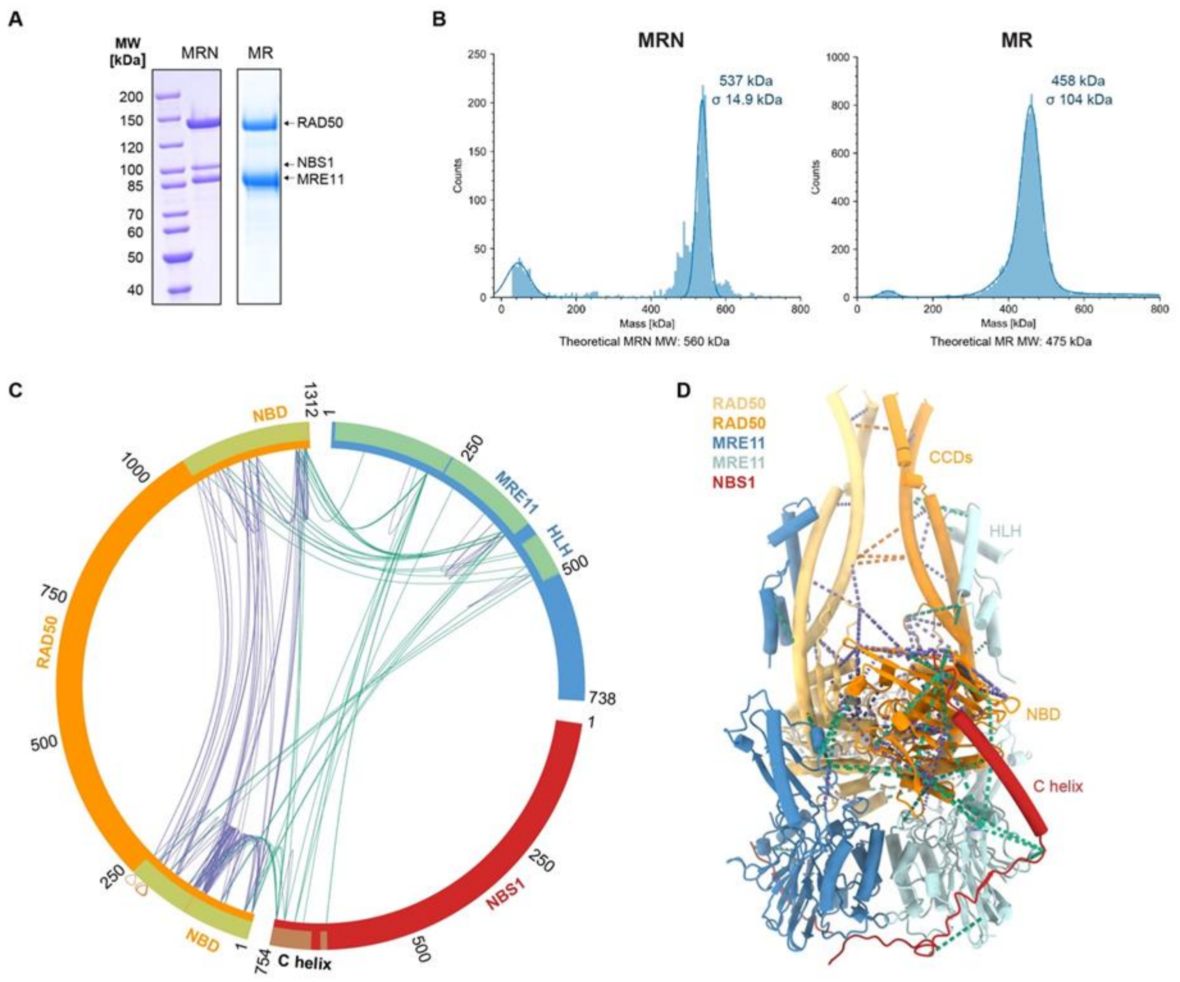


**Supplementary Fig. 1: MR(N) complex and chemical cross-linking coupled with mass spectrometry. A,** Representative SDS-PAGE analysis of purified MR/MRN complexes after gel filtration. **B,** Mass photometry histograms of the MR/MRN complexes (50 nM). **C,** XiVIEW-generated circular crosslinking map of the MRE11, RAD50, NBS1 subunits. The False Discovery Rate (FDR) was set to 1% and the crosslinking distance <30 Å. The PDB annotated regions in each subunit are highlighted in light green. Purple and green lines denote self and heteromeric crosslinks, respectively. **D,** Crosslinks mapped onto the MRN-DNA structure. DNA is not shown for better visual representation. Crosslinking-mass spectrometry data are available via ProteomeXchange with identifier PXD067324. Source data for **A and C** are provided as a Source Data file.

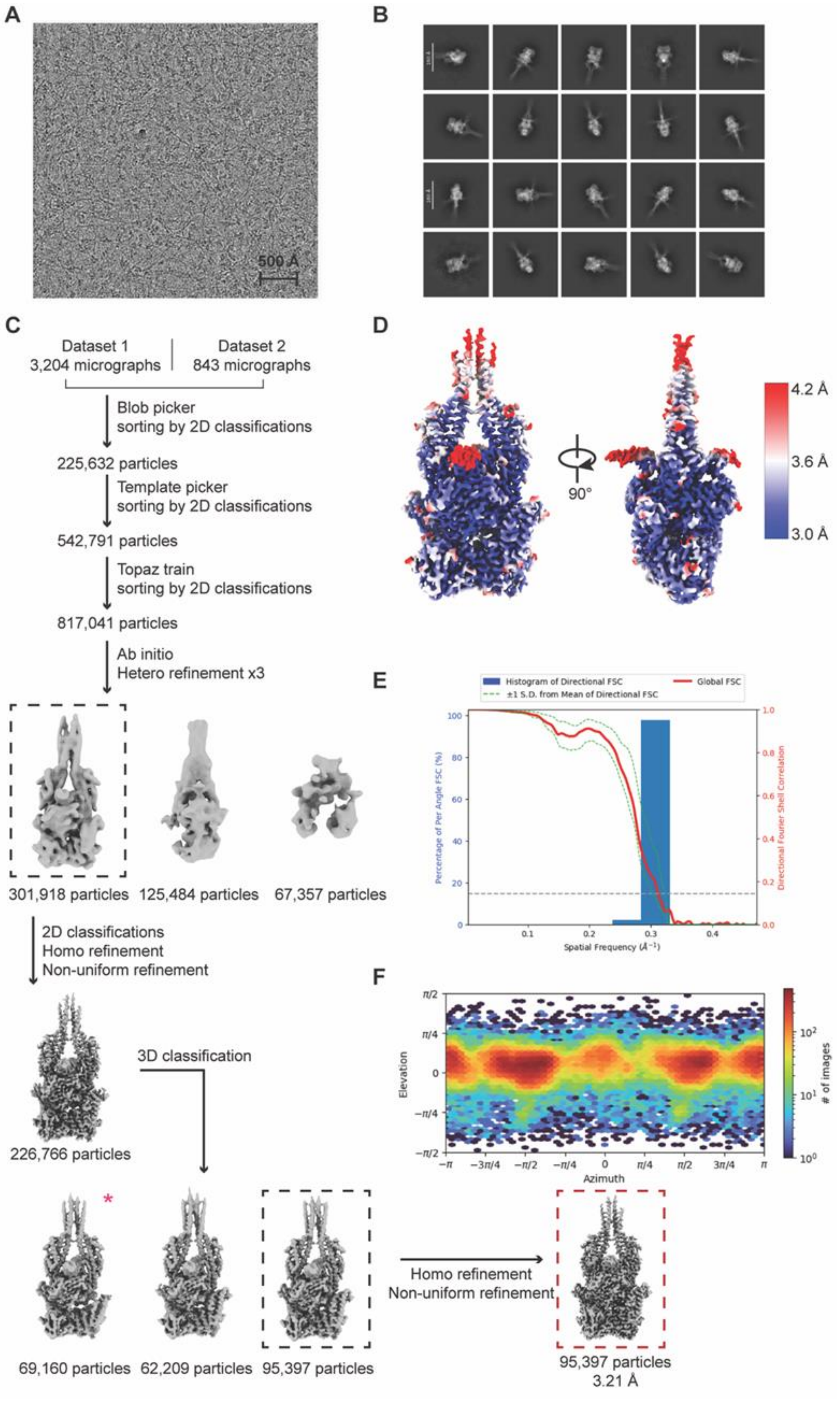
A
500 Å
B
C
Dataset 1
3,204 micrographs
Dataset 2
843 micrographs
Blob picker
sorting by 2D classifications
225,632 particles
Template picker
sorting by 2D classifications
542,791 particles
Topaz train
sorting by 2D classifications
817,041 particles
Ab initio
Hetero refinement x3
301,918 particles
125,484 particles
67,357 particles
2D classifications
Homo refinement
Non-uniform refinement
226,766 particles
3D classification
*
69,160 particles
62,209 particles
95,397 particles
Homo refinement
Non-uniform refinement
95,397 particles
3.21 Å
D
90°
4.2 Å
3.6 Å
3.0 Å
E
Histogram of Directional FSC
±1 S.D. from Mean of Directional FSC
Global FSC
Percentage of Per Angle FSC (%)
Directional Fourier Shell Correlation
Spatial Frequency (Å⁻¹)
F
Elevation
Azimuth
# of images

**Supplementary Fig. 2: Cryo-EM data analysis of MR-DNA. A,** Representative micrograph of MR-DNA among 4047 collected movies. **B,** Representative 2D classes of the particles used for the final MR-DNA reconstruction. **C,** Cryo-EM data processing workflow of MR-DNA using cryoSPARC[1]. Pink asterisk denotes the class with MRE11 detached from $RAD50^{NBD}$**. D,** Local resolution visualisation of MR-DNA calculated in cryoSPARC. Blue indicates higher resolution, red indicates lower resolution. **E,** Histogram of directional Fourier shell correlation (FSC)[2] (blue) and global FSC curve (red) of the final MR-DNA reconstruction. The spread of directional resolution values (green dashed lines) is defined as $\pm 1\sigma$. The grey dashed line shows the 0.143 cut-off criterion, indicating a nominal resolution of 3.21Å. **F,** Angular distribution of the particles used for final MR-DNA reconstruction.

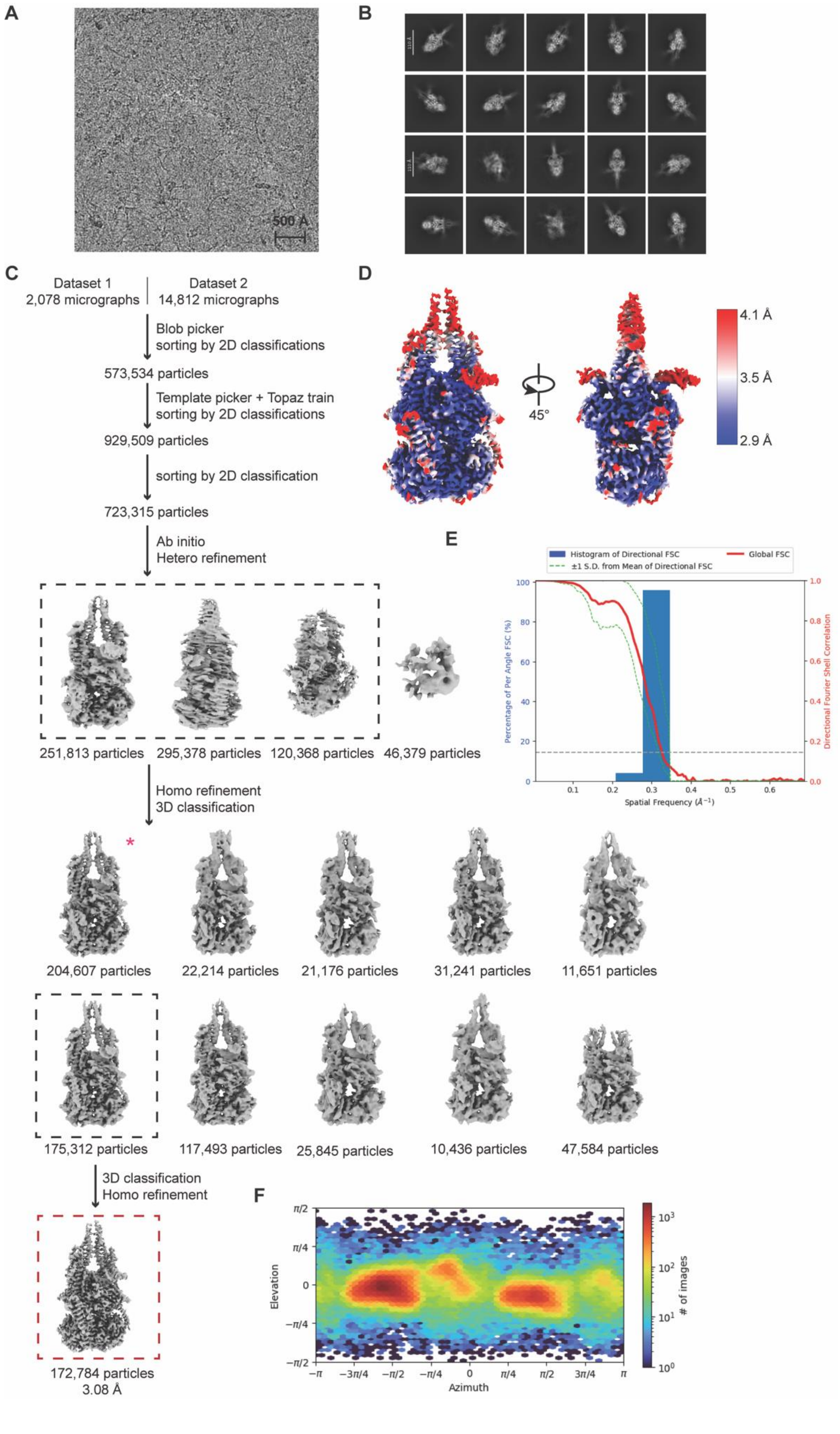

A
500 Å
B
C
Dataset 1
2,078 micrographs
Dataset 2
14,812 micrographs
Blob picker
sorting by 2D classifications
573,534 particles
Template picker + Topaz train
sorting by 2D classifications
929,509 particles
sorting by 2D classification
723,315 particles
Ab initio
Hetero refinement
251,813 particles
295,378 particles
120,368 particles
46,379 particles
Homo refinement
3D classification
204,607 particles
22,214 particles
21,176 particles
31,241 particles
11,651 particles
175,312 particles
117,493 particles
25,845 particles
10,436 particles
47,584 particles
3D classification
Homo refinement
172,784 particles
3.08 Å
D
45°
4.1 Å
3.5 Å
2.9 Å
E
Histogram of Directional FSC
±1 S.D. from Mean of Directional FSC
Global FSC
Percentage of Per Angle FSC (%)
Directional Fourier Shell Correlation
Spatial Frequency (Å$^{-1}$)
F
Elevation
Azimuth
# of images

**Supplementary Fig. 3: Cryo-EM data analysis of MRN-DNA. A,** Representative micrograph of MRN-DNA among 16,890 collected movies. **B,** Representative 2D classes of the particles used for the final MRN-DNA reconstruction. **C,** Cryo-EM data processing workflow of MRN-DNA using cryoSPARC[1]. Pink asterisk denotes the class with MRE11 detached from RAD50$^{NBD}$. **D,** Local resolution visualisation of MRN-DNA calculated in cryoSPARC. Blue indicates higher resolution, red indicates lower resolution. **E,** Histogram of directional FSC[2] (blue) and global FSC curve (red) of the final MRN-DNA reconstruction. The spread of directional resolution values (green dashed lines) is defined as $\pm$ 1σ. The grey dashed line shows the 0.143 cut-off criterion, indicating a nominal resolution of 3.08Å. **F,** Angular distribution of the particles used for final MRN-DNA reconstruction.

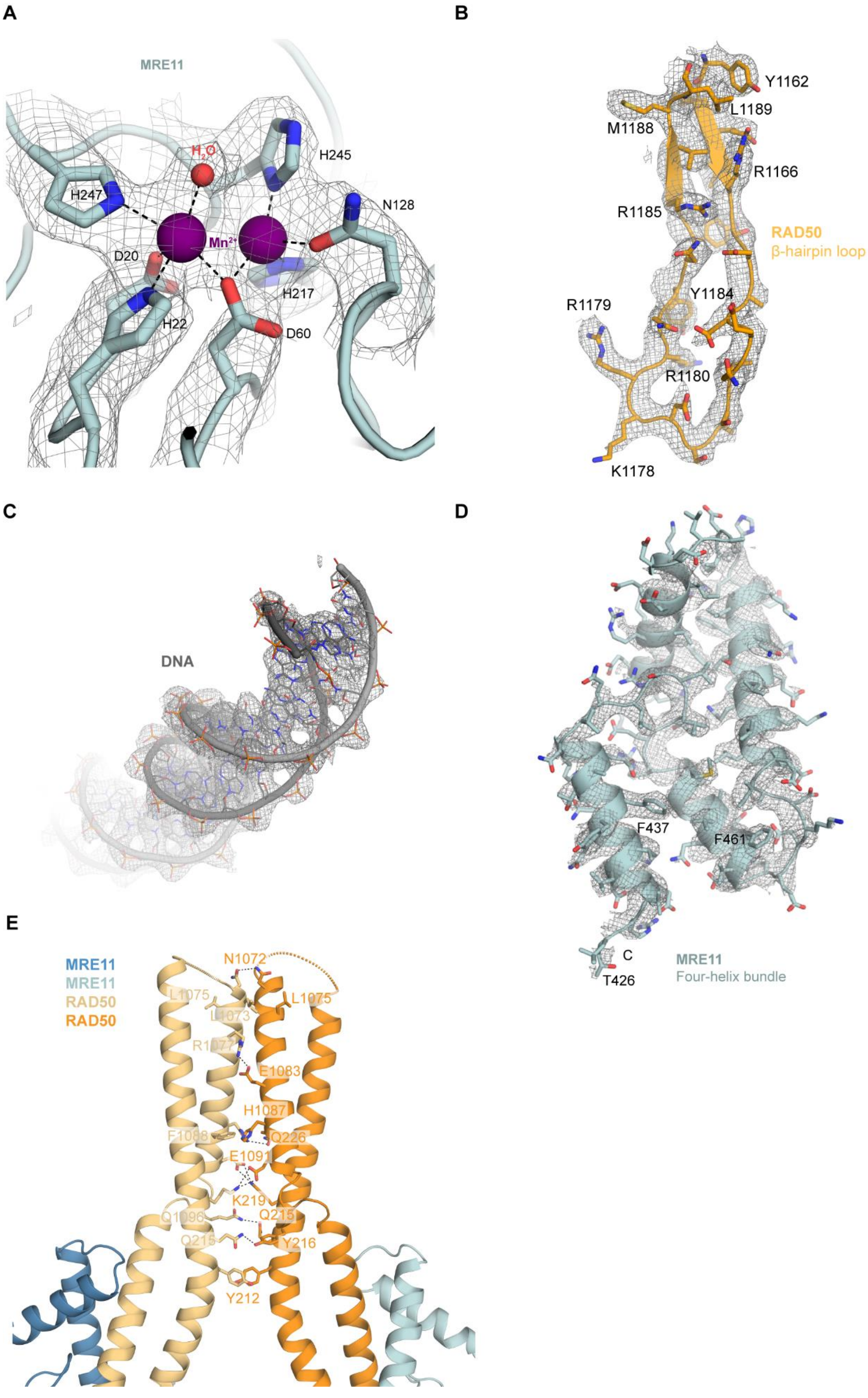
A
MRE11
H₂O
H245
H247
N128
Mn²⁺
D20
H217
H22
D60
B
Y1162
L1189
M1188
R1166
R1185
RAD50
β-hairpin loop
R1179
Y1184
R1180
K1178
C
DNA
D
F437
F461
C
T426
MRE11
Four-helix bundle
E
MRE11
MRE11
RAD50
RAD50
N1072
L1075
L1073
L1075
E1083
H1087
F1088
Q226
E1091
K219
Q1096
Q215
Q215
Y216
Y212

**Supplementary Fig. 4. Quality of cryo-EM reconstructions. A-E,** Densities for various parts of MR-TRF2$^{iDDR\text{-}Myb}$-DNA complex as indicated. Interface of RAD50 CCDs with interacting residues shown as sticks and labeled.

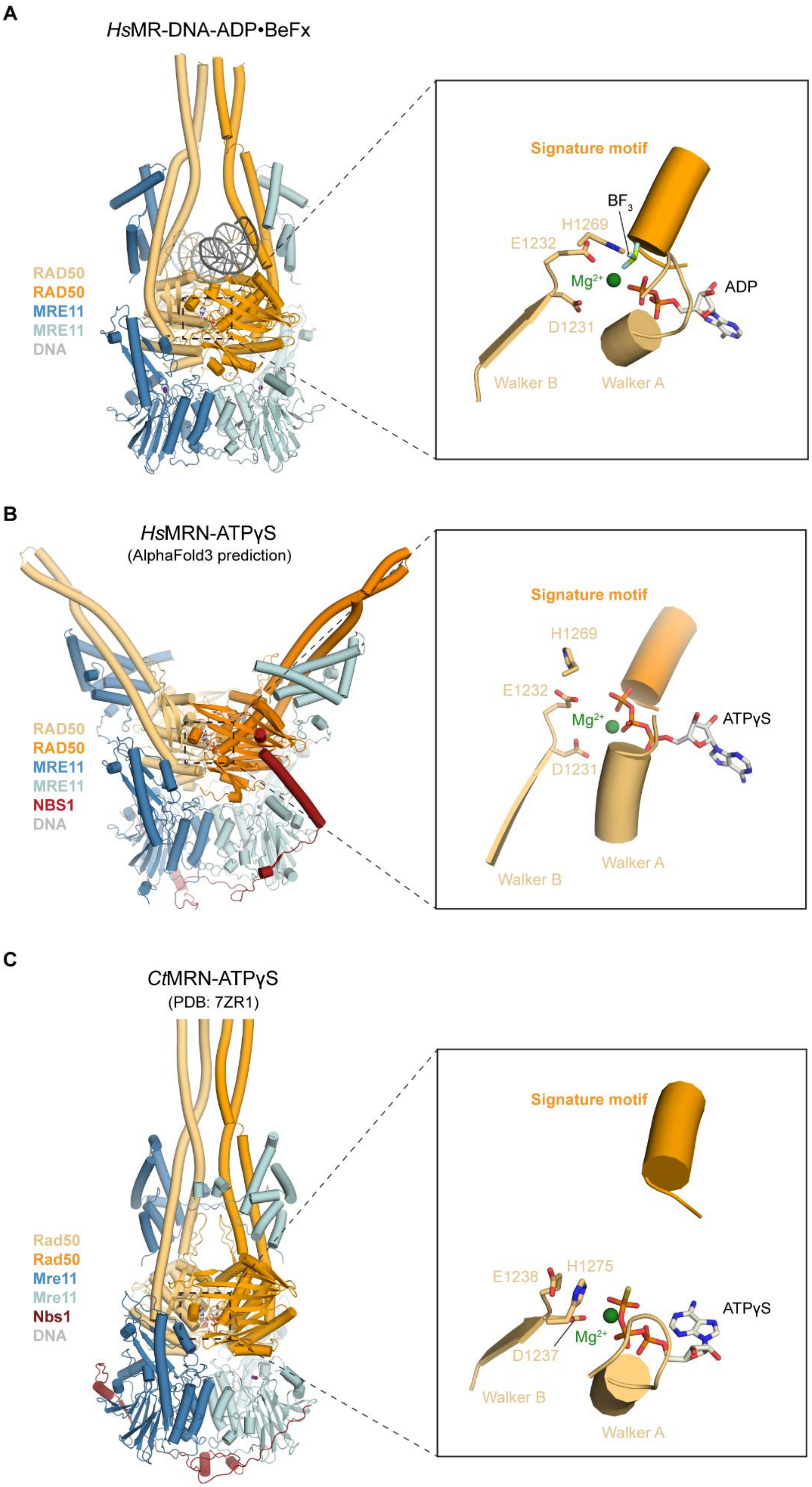
A
HsMR-DNA-ADP•BeFx
RAD50
RAD50
MRE11
MRE11
DNA
Signature motif
BF3
H1269
E1232
Mg2+
ADP
D1231
Walker B
Walker A
B
HsMRN-ATPγS
(AlphaFold3 prediction)
RAD50
RAD50
MRE11
MRE11
NBS1
DNA
Signature motif
H1269
E1232
Mg2+
ATPγS
D1231
Walker A
Walker B
C
CtMRN-ATPγS
(PDB: 7ZR1)
Rad50
Rad50
Mre11
Mre11
Nbs1
DNA
Signature motif
E1238
H1275
ATPγS
Mg2+
D1237
Walker B
Walker A

**Supplementary Fig. 5. Structural comparison of nucleotide binding pockets in different MR(N) structures. A-C,** Side-by-side comparison of MR(N) structures, emphasizing the unique structural arrangement of the signature motif surrounding RAD50's nucleotide-binding site in **A,** *Hs*MR-DNA-ADP•$BeF_x$ (current study), **B,** *Hs*MRN-ATPγS (current study) and **C,** *Ct*MRN-ATPγS (PDB: 7ZR1). Corresponding subunits were colored consistently. *Hs*: *Homo sapiens, Ct*: *Chaetomium thermophilum.*

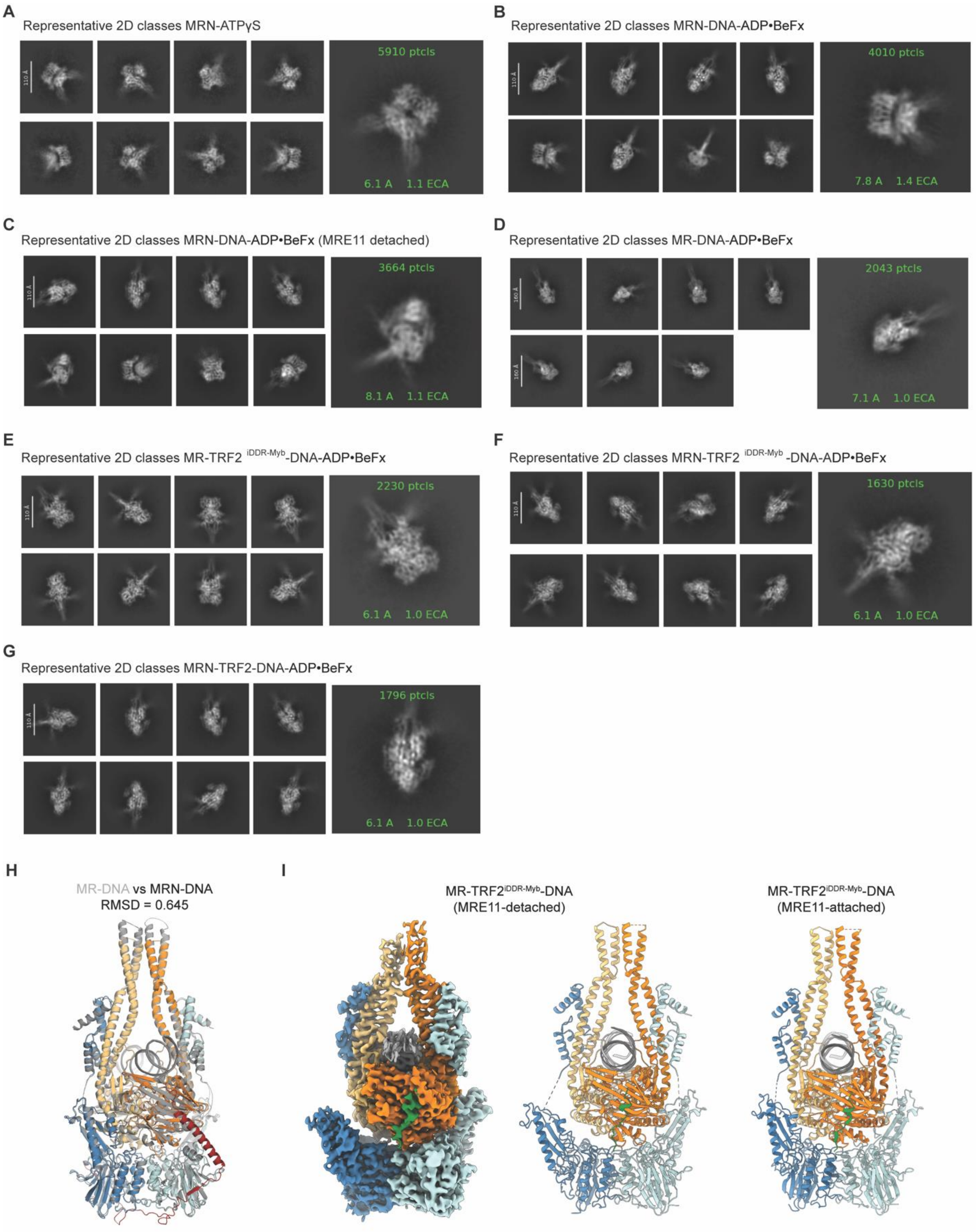
A
Representative 2D classes MRN-ATPγS
5910 ptcls
6.1 A 1.1 ECA
B
Representative 2D classes MRN-DNA-ADP•BeFx
4010 ptcls
7.8 A 1.4 ECA
C
Representative 2D classes MRN-DNA-ADP•BeFx (MRE11 detached)
3664 ptcls
8.1 A 1.1 ECA
D
Representative 2D classes MR-DNA-ADP•BeFx
2043 ptcls
7.1 A 1.0 ECA
E
Representative 2D classes MR-TRF2 iDDR-Myb-DNA-ADP•BeFx
2230 ptcls
6.1 A 1.0 ECA
F
Representative 2D classes MRN-TRF2 iDDR-Myb -DNA-ADP•BeFx
1630 ptcls
6.1 A 1.0 ECA
G
Representative 2D classes MRN-TRF2-DNA-ADP•BeFx
1796 ptcls
6.1 A 1.0 ECA
H
MR-DNA vs MRN-DNA
RMSD = 0.645
I
MR-TRF2iDDR-Myb-DNA
(MRE11-detached)
MR-TRF2iDDR-Myb-DNA
(MRE11-attached)

**Supplementary Fig. 6. MR/MRN 2D classes. A,** Representative 2D classes obtained for MRN with ATPγS. **B,** Representative 2D classes obtained for MRN with DNA and ADP•$BeF_{x.}$ The complex displayed a heterogeneity with open and closed CCDs, as well as asymmetrically bound MRE11 dimers. **C-G,** Representative 2D classes of MRE11 dimer detached as a rigid body from one $RAD50^{NBD}$. **H,** Structural superimposition of MR-DNA with MRN-DNA complex. MR-DNA structure was colored in grey for simplicity, while MRN-DNA structure was colored consistently in the manuscript. **I,** Overview of the MR-$TRF2^{iDDR\text{-}Myb}$-DNA complex structure with one partially detached MRE11 subunit (left two panels) and the side-by-side comparison to the fully attached MRE11 complex structure (rightmost panel). High resolution cryo-EM map of the partially detached MRE11 complex structure was displayed on the leftmost panel.

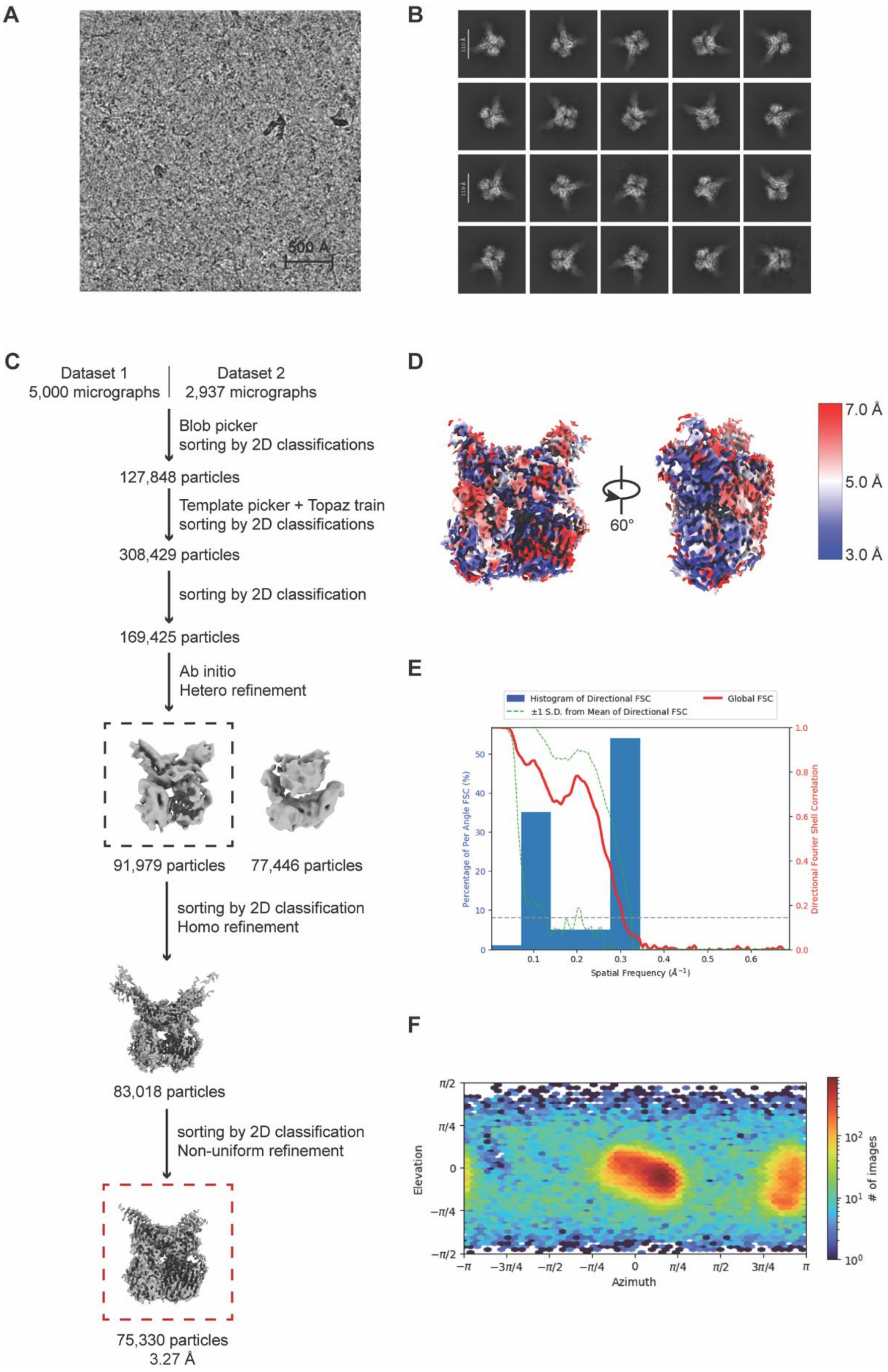
A
500 Å
B
C
Dataset 1
5,000 micrographs
Dataset 2
2,937 micrographs
Blob picker
sorting by 2D classifications
127,848 particles
Template picker + Topaz train
sorting by 2D classifications
308,429 particles
sorting by 2D classification
169,425 particles
Ab initio
Hetero refinement
91,979 particles
77,446 particles
sorting by 2D classification
Homo refinement
83,018 particles
sorting by 2D classification
Non-uniform refinement
75,330 particles
3.27 Å
D
60°
7.0 Å
5.0 Å
3.0 Å
E
Histogram of Directional FSC
±1 S.D. from Mean of Directional FSC
Global FSC
Percentage of Per Angle FSC (%)
Directional Fourier Shell Correlation
Spatial Frequency (Å⁻¹)
F
Elevation
Azimuth
# of images

**Supplementary Fig. 7: Cryo-EM data analysis of MRN-ATPγS. A,** Representative micrograph of MRN-ATPγS among 7,937 collected movies. **B,** Representative 2D classes of the particles used for the final MRN-ATPγS reconstruction. **C,** Cryo-EM data processing workflow of MRN-ATPγS using cryoSPARC[1]. **D,** Local resolution visualization of MRN-ATPγS calculated in cryoSPARC. Blue indicates higher resolution, while red indicates lower resolution. **E,** Histogram of directional Fourier shell correlation (FSC)[2] (blue) and global FSC curve (red) of the final MRN-ATPγS reconstruction. The spread of directional resolution values (green dashed lines) is defined as $\pm$ 1σ. The grey dashed line shows the 0.143 cut-off criterion, indicating a nominal resolution of 3.27 Å. **F,** Angular distribution of the particles used for final MRN-ATPγS reconstruction.

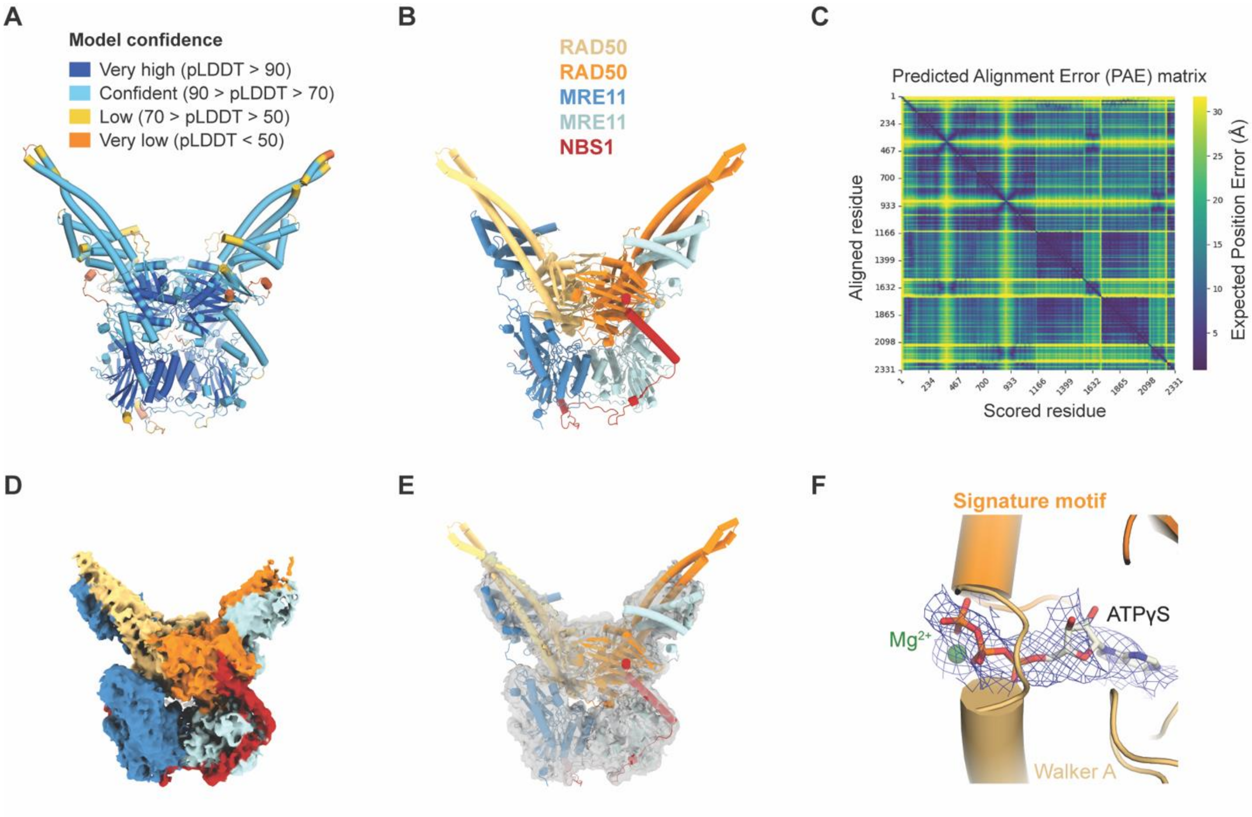


**Supplementary Fig. 8: AlphaFold3 prediction of MRN-ATP fitting into map of MRN-ATPγS. A,** AlphaFold3-predicted model of MRN-ATP colored based on pLDDT values. **B,** The same prediction colored by chain. **C,** Predicted Alignment Error (PAE) matrix of the model; darker color represents higher confidence. **D,** The cryo-EM map of MRN-ATPγS at 3.27 Å. **E,** Rigid body fitting of the AlphaFold3-predicted MRN-ATP model into the refined 3D construction map using ChimeraX[3]. **F,** Densities for ATPγS and magnesium ion in the nucleotide binding pocket of RAD50.

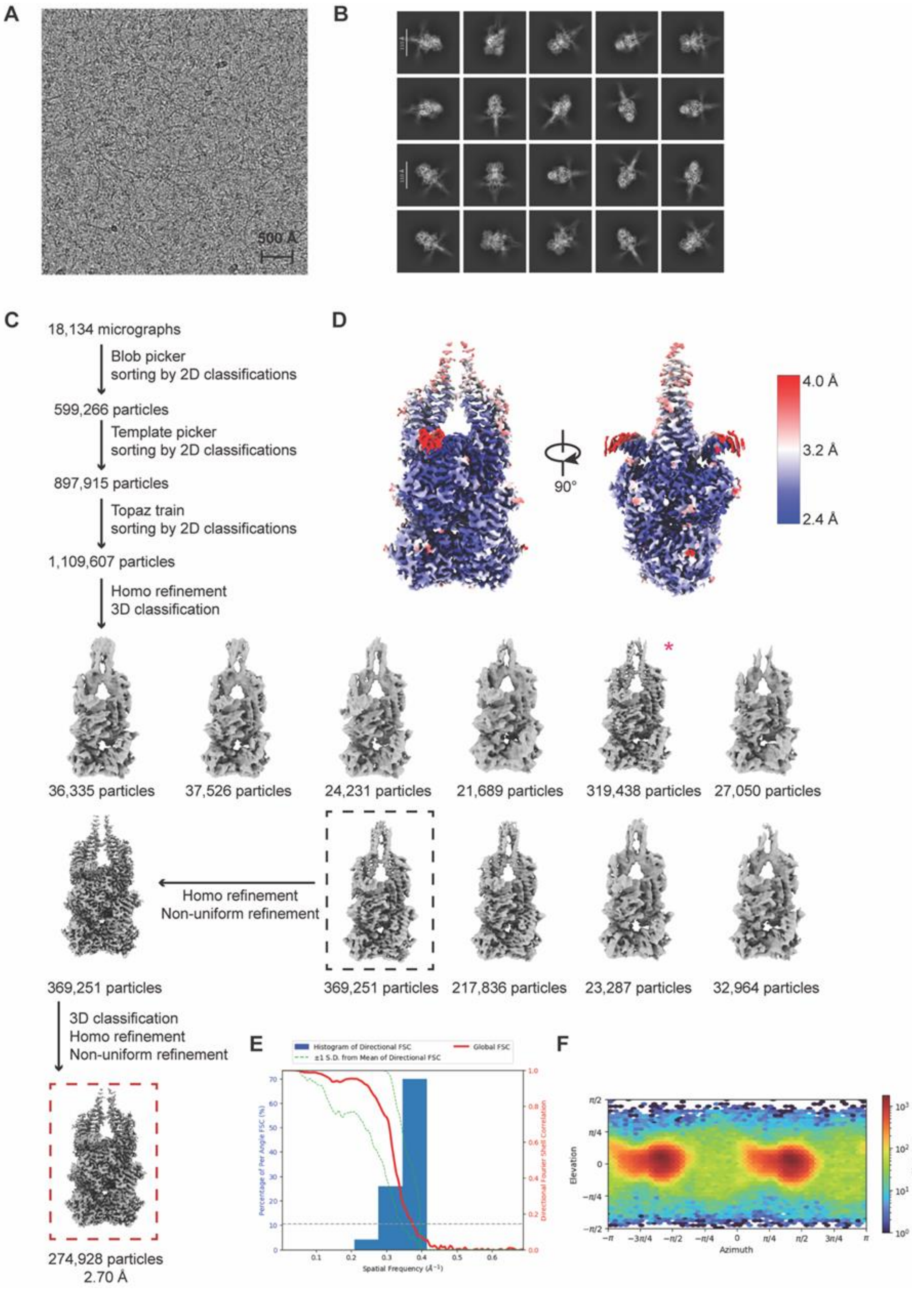
A
500 Å
B
C
18,134 micrographs
Blob picker
sorting by 2D classifications
599,266 particles
Template picker
sorting by 2D classifications
897,915 particles
Topaz train
sorting by 2D classifications
1,109,607 particles
Homo refinement
3D classification
36,335 particles
37,526 particles
24,231 particles
21,689 particles
319,438 particles
27,050 particles
Homo refinement
Non-uniform refinement
369,251 particles
369,251 particles
217,836 particles
23,287 particles
32,964 particles
3D classification
Homo refinement
Non-uniform refinement
274,928 particles
2.70 Å
D
90°
4.0 Å
3.2 Å
2.4 Å
E
Histogram of Directional FSC
±1 S.D. from Mean of Directional FSC
Global FSC
Percentage of Per Angle FSC (%)
Directional Fourier Shell Correlation
Spatial Frequency (Å⁻¹)
F
Elevation
Azimuth
# of images

**Supplementary Fig. 9: Cryo-EM data analysis of MR-TRF2$^{\text{iDDR-Myb}}$-DNA. A,** Representative micrograph of MR-TRF2$^{\text{iDDR-Myb}}$-DNA among 18,134 collected movies. **B,** Representative 2D classes of the particles used for the final MR-TRF2$^{\text{iDDR-Myb}}$-DNA reconstruction. **C,** Cryo-EM data processing workflow of MR-TRF2$^{\text{iDDR-Myb}}$-DNA using cryoSPARC[1]. Pink asterisk denotes a well-resolved class with MRE11 detached from RAD50$^{\text{NBD}}$. **D,** Local resolution visualisation of MR-TRF2$^{\text{iDDR-Myb}}$-DNA calculated in cryoSPARC. Blue indicates higher resolution, red indicates lower resolution. **E,** Histogram of directional Fourier shell correlation (FSC)[2] (blue) and global FSC curve (red) of the final MR-TRF2$^{\text{iDDR-Myb}}$-DNA reconstruction. The spread of directional resolution values (green dashed lines) is defined as $\pm$ 1σ. The grey dashed line shows the 0.143 cut-off criterion, indicating a nominal resolution of 2.7Å. **F,** Angular distribution of the particles used for final MR-TRF2$^{\text{iDDR-Myb}}$-DNA reconstruction.

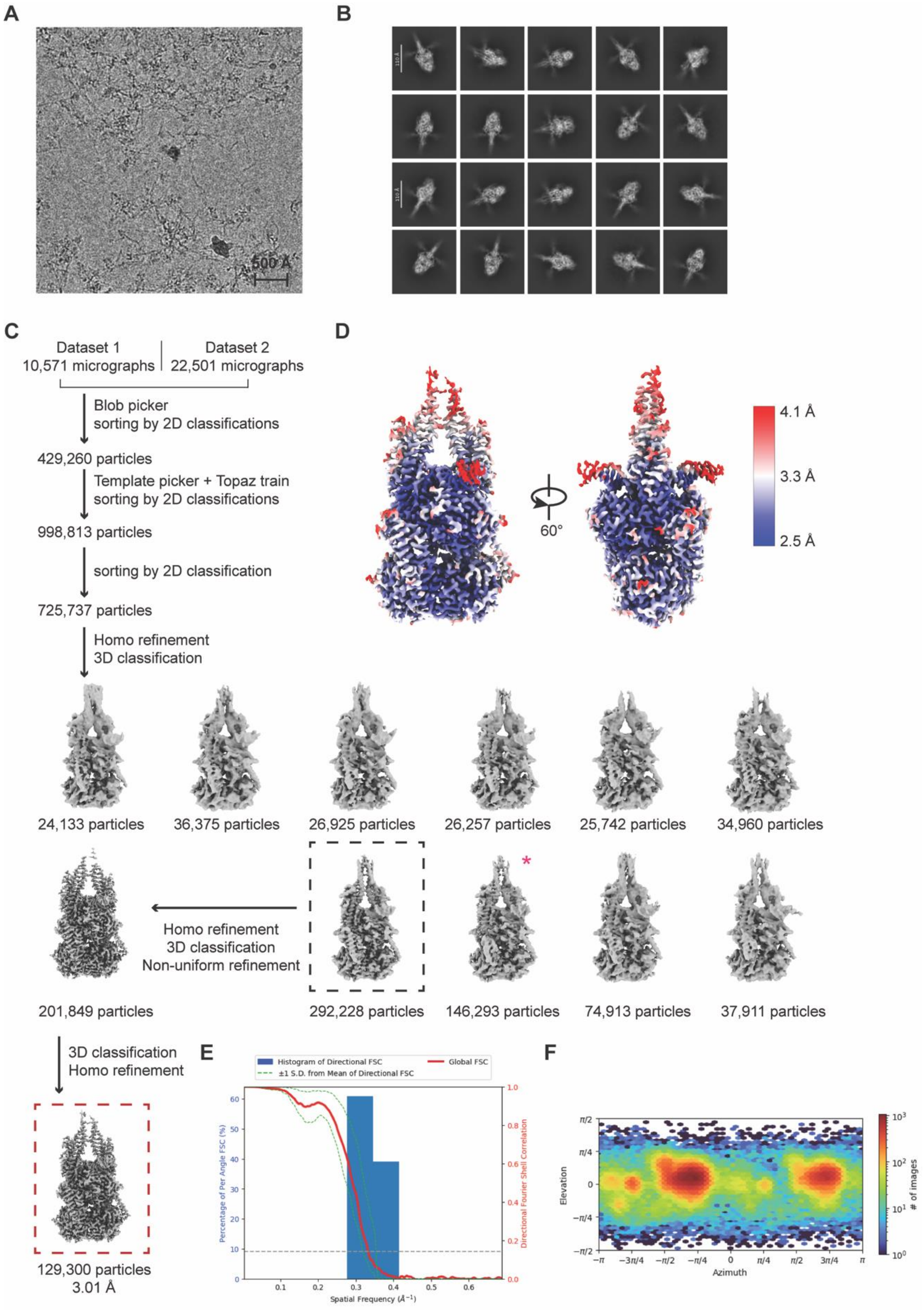

A
B
500 Å
110 Å
C
Dataset 1
10,571 micrographs
Dataset 2
22,501 micrographs
Blob picker
sorting by 2D classifications
429,260 particles
Template picker + Topaz train
sorting by 2D classifications
998,813 particles
sorting by 2D classification
725,737 particles
Homo refinement
3D classification
24,133 particles
36,375 particles
26,925 particles
26,257 particles
25,742 particles
34,960 particles
Homo refinement
3D classification
Non-uniform refinement
201,849 particles
292,228 particles
146,293 particles
74,913 particles
37,911 particles
3D classification
Homo refinement
129,300 particles
3.01 Å
D
60°
4.1 Å
3.3 Å
2.5 Å
E
Histogram of Directional FSC
±1 S.D. from Mean of Directional FSC
Global FSC
Percentage of Per Angle FSC (%)
Directional Fourier Shell Correlation
Spatial Frequency (Å⁻¹)
F
Elevation
Azimuth
# of images

**Supplementary Fig. 10: Cryo-EM data analysis of MRN-TRF2$^{\text{iDDR-Myb}}$-DNA. A,** Representative micrograph of MRN-TRF2$^{\text{iDDR-Myb}}$-DNA among 33,072 collected movies. **B,** Representative 2D classes of the particles used for the final MRN-TRF2$^{\text{iDDR-Myb}}$-DNA reconstruction. **C,** Cryo-EM data processing workflow of MRN-TRF2$^{\text{iDDR-Myb}}$-DNA using cryoSPARC[1] . Pink asterisk denotes a well-resolved class with MRE11 detached from RAD50$^{\text{NBD}}$. **D,** Local resolution visualisation of MRN-TRF2$^{\text{iDDR-Myb}}$-DNA calculated in cryoSPARC. Blue indicates higher resolution, red indicates lower resolution. **E,** Histogram of directional Fourier shell correlation (FSC)[2] (blue) and global FSC curve (red) of the final MRN-TRF2$^{\text{iDDR-Myb}}$-DNA reconstruction. The spread of directional resolution values (green dashed lines) is defined as $\pm$ 1σ. The grey dashed line shows the 0.143 cut-off criterion, indicating a nominal resolution of 3.01Å. **F,** Angular distribution of the particles used for final MRN-TRF2$^{\text{iDDR-Myb}}$-DNA reconstruction.

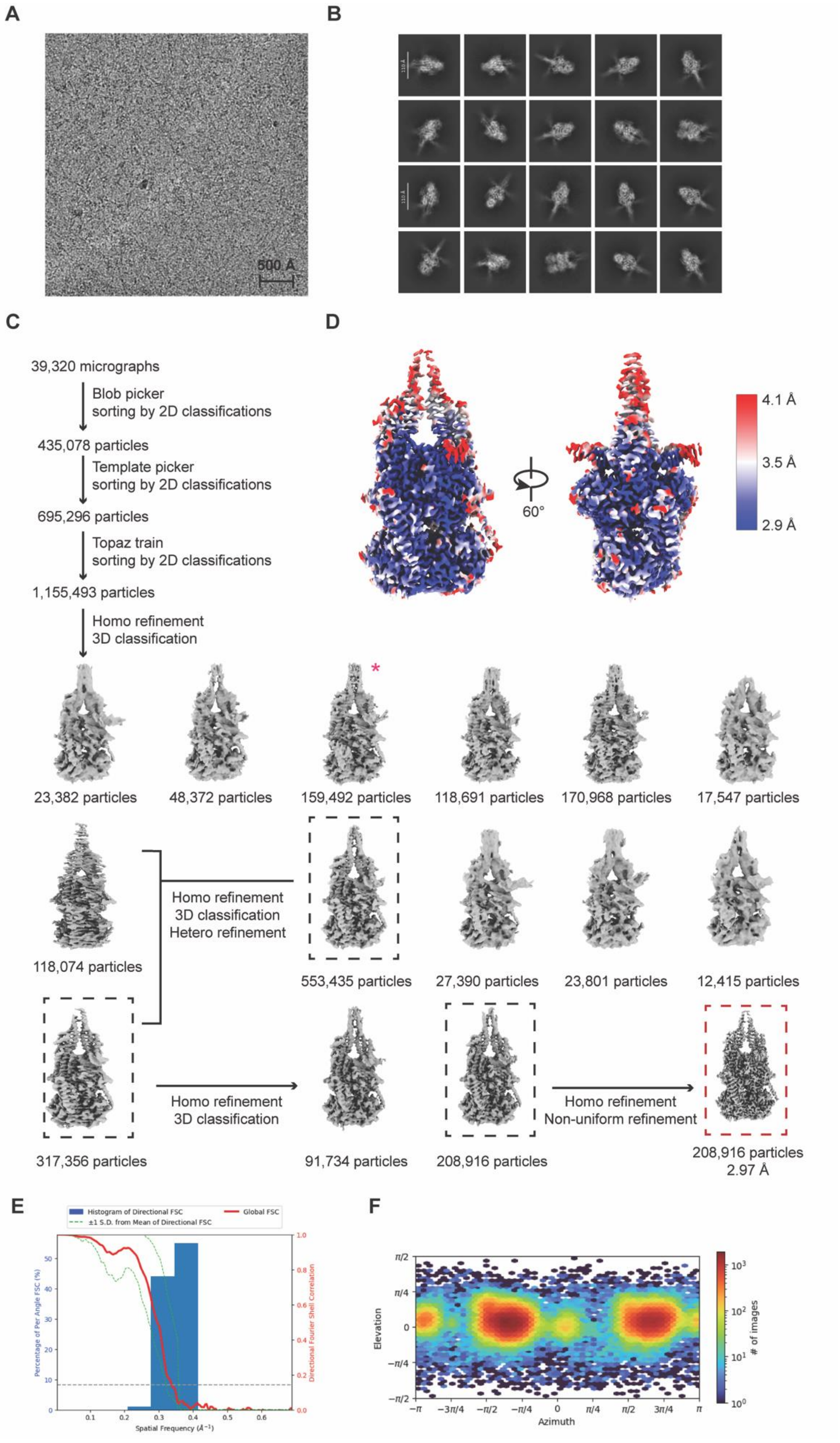

A
500 Å
B
C
39,320 micrographs
Blob picker
sorting by 2D classifications
435,078 particles
Template picker
sorting by 2D classifications
695,296 particles
Topaz train
sorting by 2D classifications
1,155,493 particles
Homo refinement
3D classification
*
23,382 particles
48,372 particles
159,492 particles
118,691 particles
170,968 particles
17,547 particles
118,074 particles
Homo refinement
3D classification
Hetero refinement
553,435 particles
27,390 particles
23,801 particles
12,415 particles
Homo refinement
3D classification
Homo refinement
Non-uniform refinement
317,356 particles
91,734 particles
208,916 particles
208,916 particles
2.97 Å
D
60°
4.1 Å
3.5 Å
2.9 Å
E
Histogram of Directional FSC
±1 S.D. from Mean of Directional FSC
Global FSC
Percentage of Per Angle FSC (%)
Directional Fourier Shell Correlation
Spatial Frequency (Å⁻¹)
F
Elevation
Azimuth
# of images

**Supplementary Fig. 11: Cryo-EM data analysis of MRN-TRF2-DNA. A,** Representative micrograph of MRN-TRF2-DNA among 39,320 collected movies. **B,** Representative 2D classes of the particles used for the final MRN-TRF2-DNA reconstruction. **C,** Cryo-EM data processing workflow of MRN-TRF2-DNA using cryoSPARC[1]. Pink asterisk denotes a well-resolved class with MRE11 detached from RAD50$^{NBD}$. **D,** Local resolution visualisation of MRN-TRF2-DNA calculated in cryoSPARC. Blue indicates higher resolution, red indicates lower resolution. **E,** Histogram of directional Fourier shell correlation (FSC)[2] (blue) and global FSC curve (red) of the final MRN-TRF2-DNA reconstruction. The spread of directional resolution values (green dashed lines) is defined as $\pm$ 1σ. The grey dashed line shows the 0.143 cut-off criterion, indicating a nominal resolution of 2.97Å. **F,** Angular distribution of the particles used for final MRN-TRF2-DNA reconstruction.

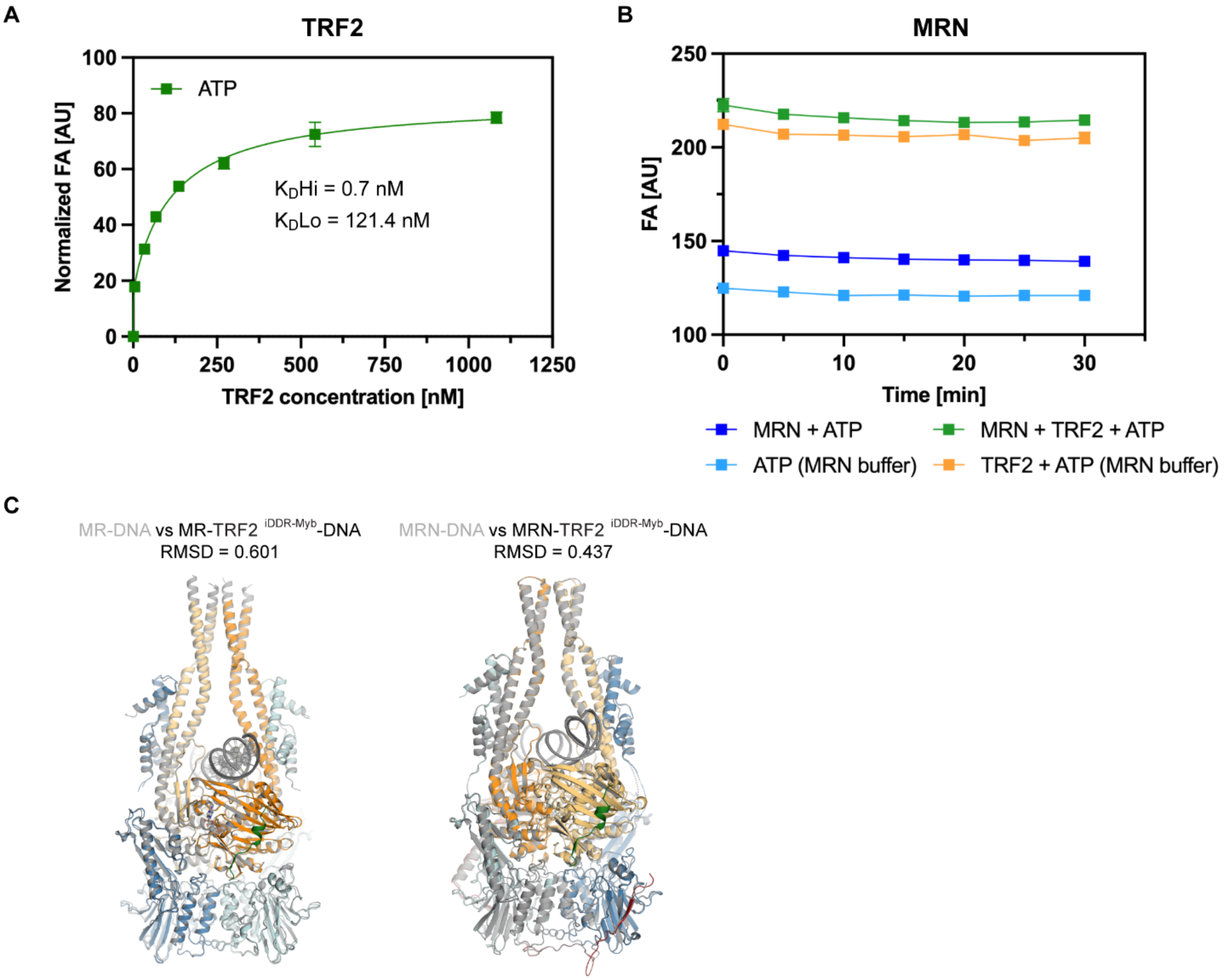


**Supplementary Fig. 12: Combined results of binding assays and structural comparison of MR(N)-DNA-(TRF2$^{iDDR-Myb}$) complexes. A,** Fluorescence anisotropy (FA)-based assay assessing full length TRF2 binding to telomeric repeat containing 64-mer DNA template in the presence of ATP. Data are presented as mean values + /− SD from three technical replicates. $K_D$ value was derived by fitting the anisotropy data to two specific binding sites with Hill slope model in Prism (GraphPad). **B,** FA signals of each titration condition kept relatively stable over the measurement time period. A concentration of 300 nM MRN and/or 1.6 μM TRF2 were used where indicated. Data are presented as mean values + /− SD from three technical replicates. **C,** Structural superimposition of MR(N)-DNA with MR(N)-TRF2$^{iDDR-Myb}$-DNA complexes. MR(N)-DNA structures were colored in grey for simplicity, while MR(N)-TRF2$^{iDDR-Myb}$-DNA complex structures were colored consistently in the manuscript. Source data for **A and B** are provided as a Source Data file.

## Supplementary Tables

### Supplementary Table 1: Chemicals and Reagents

| Chemicals and Reagents | Source | Identifier |
|---|---|---|
| ATP (99%) | Sigma-Aldrich (Merck) | Cat# A3377<br>CAS 34369-07-8 |
| ATPγS | Sigma-Aldrich (Merck) | Cat# A1388<br>CAS |
| n-Octyl-β-D-Glucopyranosid (β -OG) | Carl Roth | Cat# CN23.3 |
| BS3 (bis(sulfosuccinimidyl)suberate) | ThermoFisher | Cat #A39266 |
| $BeF_2$ (Beryllium fluoride) | Santa Cruz Biotechnology | Cat sc-263025<br>#CAS 7787-49-7 |
| BSA (recombinant albumin) | New England BioLabs | Cat# B9200S |
| Chloramphenicol | Carl Roth | Cat# Chloramphenicol<br>CAS 56-75-7 |
| DTT | Carl Roth | Cat# 6908 |
| EDTA | VWR International | Cat# 1084541000<br>CAS 6381-92-6 |
| FLAG M2 affinity resin | Sigma-Aldrich (Merck) | Cat# A2220 |
| 3x FLAG peptide | TargetMol | Cat# TP1274<br>CAS 402750-12-3 |
| Glycerol ROTIPURAN® ≥99,5 %, p.a., water free | Carl Roth | Cat# 3783<br>CAS 56-81-5 |
| HEPES<br>PUFFERAN® MIN. 99,5% P.A. | Carl Roth | Cat# 9105<br>CAS 7365-45-9 |
| Imidazole | Merck | Cat# 104716<br>CAS 288-32-4 |
| IPTG (Isopropyl-ß-D-thiogalactopyranoside) | Carl Roth | Cat# 2316.3<br>CAS 367-93-1 |
| Kanamycin (Kanamycin sulphate) | Carl Roth | Cat# T832<br>CAS 25389-94-0 |
| $MgCl_2 \cdot 6H_2O$ | Sigma-Aldrich (Merck) | Cat# 1058321000<br>CAS 7791-18-6 |
| $MnCl_2 \cdot 4H_2O$ | Carl Roth | CAS 13446-34-9 |
| NaCl | Merck | Cat# 1.06404.5000<br>CAS 7647-14-5 |

| | | |
|---|---|---|
| NADH | Carl Roth | Cat# AE12.1<br>CAS 606-68-8 |
| NaF (sodium fluoride) | Sigma-Aldrich (Merck) | Cat# S7920<br>CAS 7681-49-4 |
| $Na_4P_2O_7$ (sodiumpyrophosphat) | Sigma-Aldrich (Merck) | Cat# P8010<br>CAS 7722-88-5 |
| cOmplete EDTA-free<br>Protease Inhibitor Cocktail | Roche (Merck) | Cat# 11836170001 |
| SIGMAFAST Protease Inhibitor Cocktail Merck S8830 Tablet, EDTA free | Sigma-Aldrich (Merck) | Cat# S8830 |
| PEI MAX® - Transfection Grade Linear Polyethylenimine Hydrochloride | Polysciences | Cat# 24765<br>CAS 49553-93-7 |
| phosphoenolpyruvate (PEP) | *BioChemica*<br>Panreac AppliChem<br>ITW Reagents | Cat# A2271 |
| Phenylmethylsulfonyl fluoride (PMSF) | Sigma-Aldrich (Merck) | Cat# 52332<br>CAS 329-98-6 |
| PK/LDH (Pyruvate Kinase/Lactic Dehydrogenase) | Sigma-Aldrich (Merck) | Cat# P0294 |
| PreScission Protrase | Cytiva | Cat# 27084301 |
| Protino Ni-NTA | Machery-Nagel | Cat# 745400 |
| TCEP (Tris-(2-carboxyethyl)-phosphin Hydrochlorid) | Carl Roth | Cat# HN95.3<br>CAS 51805-45-9 |
| TRIS | Carl Roth | Cat# 4855<br>CAS 77-86-1 |
| TURBO™ DNase (2 U/µL) | Thermo-Fisher Scientific | Cat# AM2238 |
| Gibco™ Bacto™ Yeast Extract | Thermo-Fisher Scientific | Cat# 212720 |
| Bacto™ Tryptone Gibco™ | Thermo-Fisher Scientific | Cat# 211699 |

**Supplementary Table 2: Expression plasmids**

| Plasmids | Origin | Purpose |
|---|---|---|
| pACEBac1_MRE11_3C_2xFlag_RAD50 | This study | Expression in Hi5 cells |
| pACEBac1_MRE11_3C_2xFlag_RAD50_ NBS1 | This study | Expression in Hi5 cells |
| pACEMam1_MRE11$^{H129N}$_3C_2xFlag_RA D50_NBS1 | Ref.[4] | Expression in Expi293F |
| pACEMam1_MRE11_2xFlag_RAD50$^{apex}$_ NBS1 | Ref.[4] | Expression in Expi293F |
| pBig1a zz TEV YBBR RAP1 ZZ TEV TRF2 | Ahmet Yildiz, Addgene #185449, RRID:Addgene_1 85449 | Subcloning into pFastBac-MBP |
| pFastBac-MBP-3C-TRF2-$His_{10}$ | This study | Expression in Hi5 cells |
| pET47b_$His_6$-SUMO_TRF2$^{iDDR}$ (438-485) | This study | Expression in *E.coli* Rosetta |
| pET47b_$His_6$-SUMO_TRF2$^{iDDR\text{-}Myb}$ (438-542) | This study | Expression in *E.coli* Rosetta |

**Supplementary Table 3: Recombinant proteins**

| Recombinant Proteins | | Purpose |
|---|---|---|
| MRN WT (MRE11-RAD50-NBS1) | This study | EM, biochemistry |
| MR (MRE11-RAD50) | This study | EM, biochemistry |
| M$^{H129N}$RN | Ref.[4] | EM |
| MR$^{apex}$N | Ref.[4] | Crosslinking MS |
| TRF2 full-length | This study | EM |
| TRF2 (aa 438-485) (TRF2$^{iDDR}$) | This study | purification, biochemistry |
| TRF2 (aa 438-542) (TRF2$^{iDDR\text{-}Myb}$) | This study | EM, biochemistry |

**Supplementary Table 4: Deposited EM-Maps**

| **Deposited Maps** | | **Identifier** |
|---|---|---|
| MR-DNA | This study | EMDB: 52959 (3.21 Å) and PDB: 9Q9H |
| $M^{H129N}$RN-DNA | This study | EMDB: 52960 (3.08 Å) and PDB: 9Q91 |
| $M^{H129N}$RN-ATPγS | This study | EMDB: 54397 (3.27 Å) |
| MR-TRF2$^{iDDR\text{-}Myb}$ -DNA | This study | EMDB: 52962 (2.7 Å) and PDB: 9Q9K |
| MRN-TRF2$^{iDDR\text{-}Myb}$-DNA | This study | EMDB: 52961 (3.01 Å) and PDB: 9Q9J |
| MRN-TRF2-DNA | This study | EMDB: 52964 (2.97 Å) and PDB: 9Q9M |

**Supplementary Table 5: Oligonucleotides**

| **Oligonucleotides** | | **Sequence** |
|---|---|---|
| 50bp DNA fwd | This study, EM studies | TCTTTTTTTTTTTTGTTCTTTT<br>TTTTGATTCCGGTTTCTTTGA<br>AATTTTT |
| 50 bp DNA rev | This study, EM studies | AAAAATTTCAAAGAAACCGG<br>AATCAAAAAAAAGAACAAAA<br>AAAAAAAAGA |
| 80bp DNA fwd | Ref.[4] | CGGGTAGTAGATGAGCGCA<br>GGGACACCGAGGTCAAGTA<br>CATTACCCTCTCATAGGAGG<br>TGCGCTTTATCAGAAGCCAG<br>AC |
| 80bp DNA fwd | Ref.[4] | GTCTGGCTTCTGATAAAGCG<br>CACCTCCTATGAGAGGGTAA<br>TGTACTTGACCTCGGTGTCC<br>CTGCGCTCA<br>TCTACTACCCG |
| 64 bp myb DNA<br>(myb-DNA fwd 0 bp core +<br>34 bp telomeric repeat) | This study, EM studies | TCTAGGGTTAGGGTTAGATT<br>AACGCTTCTGGAGAAACTCA<br>ACGAGCTCTAACCCTAACCC<br>TAGA |
| 64 bp myb DNA<br>(myb-DNA rev 30 bp core +<br>34 bp telomeric repeat) | This study, EM studies | TCTAGGGTTAGGGTTAGAGC<br>TCGTTGAGTTTCTCCAGAAG<br>CGTTAATCTAACCCTAACCC<br>TAGA |
| MRE11_H129N<br>Forward primer | This study | CATCCACGGAAACAACGAC<br>GACCCAAC |
| MRE11_H129N<br>Reverse primer | This study | GTTGGGTCGTCGTTGTTTCC<br>GTGGATG |

**Supplementary Table 6: Commercial columns and consumables**

| **Other** | | |
|---|---|---|
| SERVAGel™ TG PRiME™ 4 - 12 % | SERVA Electrophoresis GmbH | Cat# 43288.01 |
| 1 mL HiTrap® Heparin High Performance | Cytiva | Cat# 17040601 |
| Superose® 6 Increase 10/300 GL | Cytiva | Cat# 29-0915-96 |
| Superdex® 200 Increase 10/300 GL | Cytiva | Cat# 28-9909-44 |
| Amicon Ultra-15 Centrifugal Filter Millipore Sigma 100 kDa MWCO | Sigma-Aldrich (Merck) | Cat# UFC910008 |
| Filter units Millex® glass fibre, Millex®-AP 50 | Merck-Millipore (Roth) | Cat# CY10.1 |
| Glutathione Sepharose 4 Fast Flow column | Cytiva | Cat# 17513201 |
| Greiner microplate, 384 well, flat bottom black non-binding | Greiner Bio-One | Cat# 781900 |
| Quantifoil R2/1 + 2 nm C | Quantifoil | N/A |

**Supplementary Table 7: Software and algorithms**

| **Software and algorithms** | | |
|---|---|---|
| MotionCor2 1.4.5 | Ref.[5] | https://msg.ucsf.edu/em/software/ motioncor2.html |
| Phenix 1.20.1-4487 | Ref.[6] | https://www.phenix-online.org/ |
| ChimeraX 1.6.1 | Ref.[3] | https://www.rbvi.ucsf.edu/chimerax/ |
| COOT 0.9.8.1 | Ref.[7] | https://www2.mrc-lmb.cam.ac.uk/ Personal/pemsley/coot/ |
| Prism 10.4.0 | GraphPad | https://www.graphpad.com/ |
| xiVIEW 1.0.18 | Ref.[8] | https://www.xiview.org/index.php |
| Custom-written script for FA analysis | Ref.[4] | https://zenodo.org/record/ 7353848#Y38qNnaZNnI |
| CryoSPARC 4.6.2 | Ref.[1] | https://cryosparc.com/ |
| Topaz 4.0.2 | Ref.[9,10] | https://emgweb.nysbc.org/topaz.ht |
| AlphaFold3 | Ref.[11] | https://doi.org/10.1038/s41586-024-07487-w |
| PyMOL 2.5.5 | PyMOL (The PyMOL Molecular Graphics System, v.2.5.5, Schrödinger, LLC) | https://www.pymol.org/ |
| MaxQuant 2.1.3.0 | Ref.[12] | https://www.maxquant.org/ |
| xiSEARCH 1.8.7 | Ref.[13] | https://www.rappsilberlab.org/software/xisearch/ |
| EPU 3.5.1 | ThermoFisher Scientific | |
| TEM User interface Titan 3.15.1 | ThermoFisher Scientific | |
| Digital Micrograph 3.22.1461.0 | Gatan, Inc. | |

**Supplementary Table 8: Experimental models: Cell lines and organisms**

| Cell lines | Purpose | Identifier |
|---|---|---|
| *Homo sapiens* Expi293F | Recombinant protein expression | Thermo Fisher (A14527) |
| *Escherichia coli* XL1 Blue | Cloning System, Propagation | N/A |
| *E. coli* DH10MultiBac | Bacmid generation | N/A |
| *Spodoptera frugiperda* (Sf21) | Virus production | Thermo Fisher (11497013) |
| *Trichoplusia ni* insect cells (Hi5) | Recombinant protein expression | Invitrogen (B85502) |
| *E.coli* BL21 Rosetta | Recombinant protein expression | N/A |

**Supplementary References**


1. Punjani, A., Rubinstein, J.L., Fleet, D.J., and Brubaker, M.A. (2017). cryoSPARC: algorithms for rapid unsupervised cryo-EM structure determination. Nat Methods *14*, 290-296. 10.1038/nmeth.4169.
2. Tan, Y.Z., Baldwin, P.R., Davis, J.H., Williamson, J.R., Potter, C.S., Carragher, B., and Lyumkis, D. (2017). Addressing preferred specimen orientation in single-particle cryo-EM through tilting. Nat Methods *14*, 793-796. 10.1038/nmeth.4347.
3. Goddard, T.D., Huang, C.C., Meng, E.C., Pettersen, E.F., Couch, G.S., Morris, J.H., and Ferrin, T.E. (2017). UCSF ChimeraX: Meeting modern challenges in visualization and analysis. Protein Sci. 10.1002/pro.3235.
4. Rotheneder, M., Stakyte, K., van de Logt, E., Bartho, J.D., Lammens, K., Fan, Y., Alt, A., Kessler, B., Jung, C., Roos, W.P., et al. (2023). Cryo-EM structure of the Mre11-Rad50-Nbs1 complex reveals the molecular mechanism of scaffolding functions. Mol Cell *83*, 167-185 e169. 10.1016/j.molcel.2022.12.003.
5. Zheng, S.Q., Palovcak, E., Armache, J.P., Verba, K.A., Cheng, Y., and Agard, D.A. (2017). MotionCor2: anisotropic correction of beam-induced motion for improved cryo-electron microscopy. Nat Methods *14*, 331-332. 10.1038/nmeth.4193.
6. Afonine, P.V., Poon, B.K., Read, R.J., Sobolev, O.V., Terwilliger, T.C., Urzhumtsev, A., and Adams, P.D. (2018). Real-space refinement in PHENIX for cryo-EM and crystallography. Acta Crystallogr D Struct Biol *74*, 531-544. 10.1107/S2059798318006551.
7. Emsley, P., and Cowtan, K. (2004). Coot: model-building tools for molecular graphics. Acta Crystallogr D Biol Crystallogr *60*, 2126-2132. 10.1107/S0907444904019158.
8. Combe, C.W., Graham, M., Kolbowski, L., Fischer, L., and Rappsilber, J. (2024). xiVIEW: Visualisation of Crosslinking Mass Spectrometry Data. J Mol Biol *436*, 168656. 10.1016/j.jmb.2024.168656.
9. Bepler, T., Kelley, K., Noble, A.J., and Berger, B. (2020). Topaz-Denoise: general deep denoising models for cryoEM and cryoET. Nat Commun *11*, 5208. 10.1038/s41467-020-18952-1.

10. Bepler, T., Morin, A., Rapp, M., Brasch, J., Shapiro, L., Noble, A.J., and Berger, B. (2019). Positive-unlabeled convolutional neural networks for particle picking in cryo-electron micrographs. Nature Methods *16*, 1153-1160. 10.1038/s41592-019-0575-8.
11. Abramson, J., Adler, J., Dunger, J., Evans, R., Green, T., Pritzel, A., Ronneberger, O., Willmore, L., Ballard, A.J., Bambrick, J., et al. (2024). Accurate structure prediction of biomolecular interactions with AlphaFold 3. Nature *630*, 493-500. 10.1038/s41586-024-07487-w.
12. Cox, J., and Mann, M. (2008). MaxQuant enables high peptide identification rates, individualized p.p.b.-range mass accuracies and proteome-wide protein quantification. Nat Biotechnol *26*, 1367-1372. 10.1038/nbt.1511.
13. Mendes, M.L., Fischer, L., Chen, Z.A., Barbon, M., O'Reilly, F.J., Giese, S.H., Bohlke-Schneider, M., Belsom, A., Dau, T., Combe, C.W., et al. (2019). An integrated workflow for crosslinking mass spectrometry. Mol Syst Biol *15*, e8994. 10.15252/msb.20198994.
