# Supplementary material for: Structural basis for DNA break sensing by human MRE11-RAD50-NBS1 and its regulation by telomeric factor TRF2
Source: Nat Commun. 2025 Sep 18;16:8320. doi: 10.1038/s41467-025-64082-x (PMC12446458; doi:10.1038/s41467-025-64082-x)
Supplement: Supplementary file 2 — Reporting Summary [file 41467_2025_64082_MOESM2_ESM.pdf]

nature portfolio

Corresponding author(s): Karl-Peter Hopfner

Last updated by author(s): Aug 18, 2025

Reporting Summary

Nature Portfolio wishes to improve the reproducibility of the work that we publish. This form provides structure for consistency and transparency in reporting. For further information on Nature Portfolio policies, see our Editorial Policies and the Editorial Policy Checklist.

Statistics

For all statistical analyses, confirm that the following items are present in the figure legend, table legend, main text, or Methods section.

n/a	Confirmed	
☐	☒	The exact sample size (*n*) for each experimental group/condition, given as a discrete number and unit of measurement
☐	☒	A statement on whether measurements were taken from distinct samples or whether the same sample was measured repeatedly
☒	☐	The statistical test(s) used AND whether they are one- or two-sided<br>*Only common tests should be described solely by name; describe more complex techniques in the Methods section.*
☒	☐	A description of all covariates tested
☒	☐	A description of any assumptions or corrections, such as tests of normality and adjustment for multiple comparisons
☐	☒	A full description of the statistical parameters including central tendency (e.g. means) or other basic estimates (e.g. regression coefficient) AND variation (e.g. standard deviation) or associated estimates of uncertainty (e.g. confidence intervals)
☒	☐	For null hypothesis testing, the test statistic (e.g. *F*, *t*, *r*) with confidence intervals, effect sizes, degrees of freedom and *P* value noted<br>*Give P values as exact values whenever suitable.*
☒	☐	For Bayesian analysis, information on the choice of priors and Markov chain Monte Carlo settings
☒	☐	For hierarchical and complex designs, identification of the appropriate level for tests and full reporting of outcomes
☒	☐	Estimates of effect sizes (e.g. Cohen's *d*, Pearson's *r*), indicating how they were calculated

Our web collection on statistics for biologists contains articles on many of the points above.

Software and code

Policy information about availability of computer code

Data collection	EPU 3.5.1, TEM User interface Titan 3.15.1, Digital Micrograph 3.22.146
Data analysis	MotionCor2 1.4.5, Phenix 1.20.1-4487, ChimeraX 1.6.1, COOT 0.9.8.1, Prism 10.4.0, xiVIEW 1.0.18, CryoSPARC 4.6.2, Topaz 4.0.2, AlphaFold3, PyMOL 2.5.5, MaxQuant 2.1.3.0, xiSEARCH 1.8.7

For manuscripts utilizing custom algorithms or software that are central to the research but not yet described in published literature, software must be made available to editors and reviewers. We strongly encourage code deposition in a community repository (e.g. GitHub). See the Nature Portfolio guidelines for submitting code & software for further information.

Data

Policy information about availability of data

All manuscripts must include a data availability statement. This statement should provide the following information, where applicable:

- Accession codes, unique identifiers, or web links for publicly available datasets
- A description of any restrictions on data availability
- For clinical datasets or third party data, please ensure that the statement adheres to our policy

Cryo-EM data generated in this study were deposited as coordinate files in the Protein Data Bank (https://www.rcsb.org) and reconstructions in the Electron Microscopy Data Bank (https://www.ebi.ac.uk/pdbe/emdb/) under the following accession codes for each structure:
MR-DNA: EMDB: 52959 (3.21 Å) and PDB: 9Q9H. MRN-DNA complex: EMDB: 52960 (3.08 Å) and PDB: 9Q9I. MR-TRF2iDDR-Myb-DNA complex: EMDB: 52962 (2.7 Å)

and PDB: 9Q9K. MRN-TRF2iDDR-Myb-DNA complex: EMDB: 52961 (3.01 Å) and PDB: 9Q9J.
MRN-TRF2-DNA complex: EMDB: 52964 (2.97 Å) and PDB: 9Q9M. MH129NRN-ATPγS EMDB: 54397 (3.27 Å) . Crosslinking-mass spectrometry data are available via ProteomeXchange with identifier PXD067324. Plasmids and materials that were generated in this study as well as further information requests are available from the lead contact Karl-Peter Hopfner (karlpeter.hopfner@lmu.de).

Research involving human participants, their data, or biological material

Policy information about studies with human participants or human data. See also policy information about sex, gender (identity/presentation), and sexual orientation and race, ethnicity and racism.

Reporting on sex and gender	This study did not include human participants. This section is not applicable.
Reporting on race, ethnicity, or other socially relevant groupings	This study did not include human participants. This section is not applicable.
Population characteristics	This study did not include human participants. This section is not applicable.
Recruitment	This study did not include human participants. This section is not applicable.
Ethics oversight	This study did not include human participants. This section is not applicable.

Note that full information on the approval of the study protocol must also be provided in the manuscript.

Field-specific reporting

Please select the one below that is the best fit for your research. If you are not sure, read the appropriate sections before making your selection.

☒ Life sciences ☐ Behavioural & social sciences ☐ Ecological, evolutionary & environmental sciences

For a reference copy of the document with all sections, see nature.com/documents/nr-reporting-summary-flat.pdf

Life sciences study design

All studies must disclose on these points even when the disclosure is negative.

Sample size	Sample sizes were determined based on previous studies. No statistical methods were used to predetermine sample size. Replicate numbers were chosen to obtain statistical significance and reproducibility. Sample size of Cryo-EM datasets was based on number of images and particles that lead to high resolution reconstructions.
Data exclusions	No data were excluded.
Replication	Experiments that led to quantitative conclusions were performed in independent replicates as described.
Randomization	Randomization of statistical calculations was not required in this study. In cryoSPARC, particles were randomly assigned to half-maps for resolution determination.
Blinding	Blinding was not perfomed.

Reporting for specific materials, systems and methods

We require information from authors about some types of materials, experimental systems and methods used in many studies. Here, indicate whether each material, system or method listed is relevant to your study. If you are not sure if a list item applies to your research, read the appropriate section before selecting a response.

Materials & experimental systems

n/a	Involved in the study	
☒	☐	Antibodies
☐	☒	Eukaryotic cell lines
☒	☐	Palaeontology and archaeology
☒	☐	Animals and other organisms
☒	☐	Clinical data
☒	☐	Dual use research of concern
☒	☐	Plants

Methods

n/a	Involved in the study	
☒	☐	ChIP-seq
☒	☐	Flow cytometry
☒	☐	MRI-based neuroimaging

Eukaryotic cell lines

Policy information about cell lines and Sex and Gender in Research

Cell line source(s)	Spodoptera frugiperda Sf21 insect cells (Thermo Fisher, 11497013)<br>Trichoplusia ni High Five insect cells (Invitrogen, B85502), Homo sapiens Expi293F (Thermo Fisher A14527).
Authentication	No methods were used for authentication.
Mycoplasma contamination	Expi293F cell line was tested for mycoplasma contamination (negative). Other cell lines were not tested.
Commonly misidentified lines<br>(See ICLAC register)	No commonly misidentified cell lines were used.

Plants

Seed stocks	Not used.
Novel plant genotypes	Not used.
Authentication	Not used.
